# Construction of saturated Tn-Seq libraries of *Brucella abortus* S19 for transposon insertion and effective density analysis across stress conditions

**DOI:** 10.1128/mra.01099-25

**Published:** 2026-04-15

**Authors:** Emily Knebel, Gayatri Sharma, Patrick Curtis, Pamela J. B. Brown

**Affiliations:** 1 Department of Veterinary Pathobiology, University of Missouri–Columbia 14716, Columbia, Missouri, USA; 2 Department of Biology, University of Mississippi 8083, University, Mississippi, USA; 3 Division of Biological Sciences, University of Missouri–Columbia 14716, Columbia, Missouri, USA; Indiana University Bloomington, Bloomington, Indiana, USA

**Keywords:** Tn-Seq, *Brucella*, stress responses

## Abstract

*Brucella abortus* is a globally significant zoonotic pathogen. To explore the functional relevance of its genes in acidic, oxidative, and antimicrobial stress conditions, we constructed high-density transposon sequencing libraries in the attenuated *B. abortus* S19 strain. Here, we present the results and analysis of transposon insertion frequencies and effective densities.

## ANNOUNCEMENT

*Brucella abortus* is a facultatively intracellular zoonotic pathogen that must be highly adaptable to stressful intracellular environments, including acidity, oxidative stress, and antimicrobial production (1–4). This study utilizes next-generation sequencing of transposon insertion mutant libraries (Tn-Seq [5, 6]) to determine essential and influential genes in the attenuated *B. abortus* S19 genome and compare the richness and abundance of transposon insertions recovered from mutant pools exposed to an array of conditions (Table 1).

**TABLE 1:** Summary of *Brucella abortus* S19 TnSeq libraries

	Library 1	Library 2	Library 3	Library 4	Library 5	Library 6	Library 7
Growth and stress conditions							
Growth conditions^*a*^	2YT Liquid, 15 h	2YT Liquid, 14 h	2YT Liquid, 17 h	2YT Plate, 48 h	2YT Plate, 48 h	2YT Plate, 48 h	2YT Plate, 48 h
Stress conditions	Not applicable	pH 4.5^*b*^, 2 h	1.25 mM H_2_O_2,_ 1 h	Not applicable	25 μg/mL Colistin^*c*^	0.3 μg/mL Ampicillin	100 μg/mL Cefsulodin
Library sequencing metrics							
Tn-containing reads mapped to *B. abortus* S19^*d*^	40,575,958	28,790,726	35,263,787	6,257,371	19,003,077	27,624,241	29,924,858
Coverage^*e*^	1,124.39	797.81	977.18	173.40	526.59	765.49	829.24
Max # of Tn reads/ORFs^*f*^	2,282,625	2,360,933	3,536,248	685,152	1,488,431	1,907,135	2,119,624
Average Tn reads/ORF	11,974.18	8,540.44	10,446.11	1,890.83	5,683.24	8,267.12	8,826.69
Total unique Tn insertions/ORFs	556,658	552,615	584,475	257,101	436,913	534,179	511,887
Max # of unique Tn insertions/ORF	2,181	2,317	2,633	1,179	1,618	1,925	1,816
Average unique Tn insertions/ORF	175.16	173.89	183.91	80.90	137.48	168.09	161.07

^*a*^ All cultures grown at 37°C. All libraries had been previously collected from 2 days of growth on 2YT with kanamycin 50 µg/mL (Kan50). Each library utilized scraped pools of seven 150 mm plates resuspended in 2YT and represents one biological replicate. Total mutant counts of each library were approximately 67,800–220,000, from the master library containing a minimum of 1.22 million mutants. Libraries 1–3 were diluted 1:2,000 in 500 mL flasks of 2YT Kan50 and grown overnight until OD_600_ values doubled two to three times. Cells were harvested by centrifugation (7000 rpm, 15 min) and exposed to the indicated stressors. Library 4 was immediately prepared for DNA extraction after initial 48 h growth on 2YT Kan50. Libraries 5–7 were diluted 1:100 and plated on 150 mm 2YT plates containing the indicated stressors. All plates were incubated for 2 days, then scraped, pooled by condition, and resuspended for DNA extraction.

^*b*^ 2YT media buffered with acetic acid.

^*c*^ All materials prepared in glass (other than pipet tips and petri dishes) to minimize plastic binding.

^*d*^ Reads were filtered for the transposon specific primer sequence and then further trimmed to remove the first 26 bp and mapped to NCBI reference genomes for *B. abortus* S19 chromosomes I (NC_010742.1) and II (NC_010740.1).

^*e*^ Coverage is calculated as [[read length 91 (150 - Tn primer sequence and first 26 bp)] × number of reads]/3283936 bp (*B. abortus* S19 genome size).

^*f*^ ORF, open reading frames including tRNAs, rRNAs, and riboswitches.

The complete methods for Tn-seq library construction can be found at https://dx.doi.org/10.17504/protocols.io.n92ldr478g5b/v1. Briefly, matings were performed by mixing overnight cultures of the *Escherichia coli* diaminopimelic acid (DAP) auxotroph MFD *pir* (7, 8) containing pXMCS-2-miniTn5-Km (9) and *B. abortus* S19 (10) on 0.22 µm polyethylsulfone filters on 2× Yeast-Tryptone (2YT) plates containing 0.3 mM DAP. Conjugants were pooled in 2YT containing 50 µg/mL kanamycin (Kan50) and enumerated. The master TnSeq library contained 1.22–2.35 × 10^6^ Tn mutants/mL and was collected on 2YT Kan50 plates after 2 days of growth, then resuspensions were divided into seven mutant pools comprising libraries 1–7, which were then treated as described in Table 1 and harvested for phenol-chloroform DNA extraction following the recommended protocol (11).

**Fig 1:**
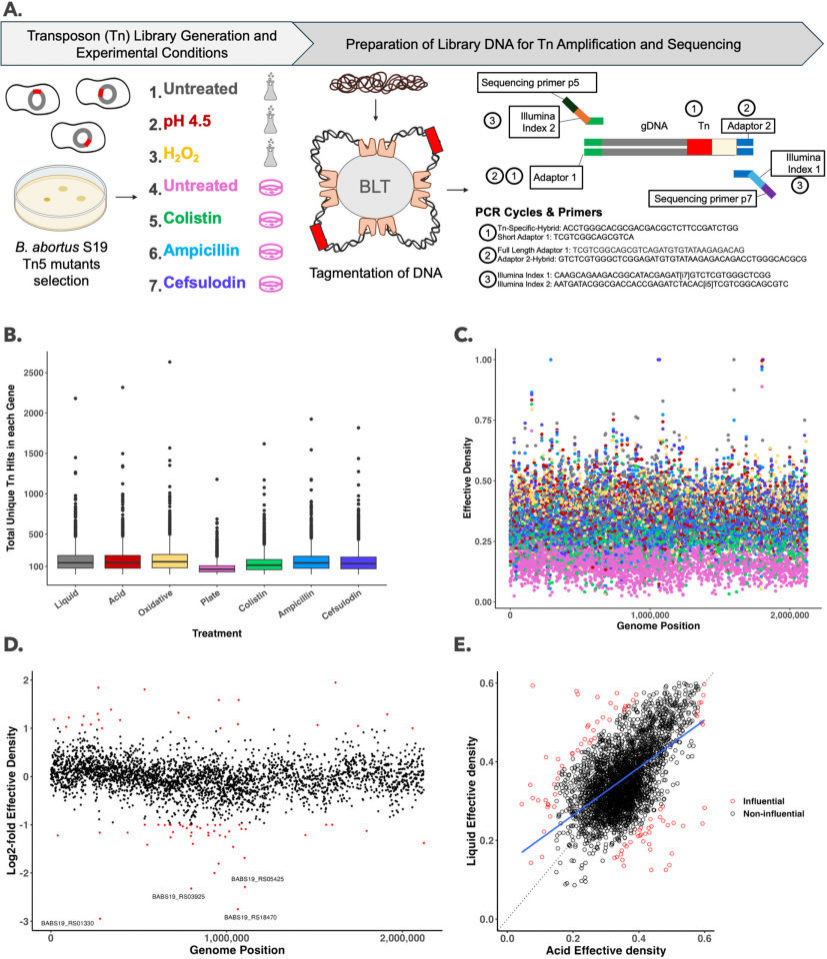
Overview of *B. abortus* S19 Tn-Seq library construction and analysis pipelines identifies conditionally essential, advantageous, and influential genes. (**A**) Schematic of transposon (Tn) mutant library construction. *B. abortus* S19 Tn5 (red bar depicted in circular genome) insertion mutants selected after conjugation, pooled, and split into seven transposon mutant libraries consisting of different growth conditions. Bacterial DNA extracted after each growth condition and tagmented with bead-linked transposomes (BLTs) with simultaneous adaptor ligation. The red box on the DNA represents the Tn5 transposon sequence. Three nested PCR reactions using Illumina DNA Prep Kit with custom primers to (i) enrich Tn sequences and associated genomic DNA (gDNA) using Tn5 and Adaptor 1-specific primers, (ii) restore full adaptor sequences with Adaptor 1 and 2-specific primers, then (iii) add unique index pairs and standard Illumina sequencing primers. Complete experimental methods can be found on protocols.io as referenced in Data Availability. (**B**) Box and whisker plots display the total unique Tn insertion hits for each gene in the *B. ab*o*rtus* S19 genome for each condition. Mean insertion density per gene varied by condition with the lowest in the Plate condition. (**C**) Scatterplot of the calculated Tn effective density for each gene in all seven libraries displayed by genomic base pair position. Treatment colors are as shown in panel B. Lower effective density values reflect the lower abundance of Tn insertions within each gene accounting for gene size. (**D**) Representative scatterplot comparing the log2 fold difference in effective density in acid-treated and untreated *B. abortus* S19 TnSeq libraries for each gene. Genes whose effective densities are labeled in red are either advantageous (log2 fold values > 1) or essential (log2 fold values < −1) for survival in acid. (**E**) Representative linear regression plot of Tn effective density for each gene in acid-treated and untreated *B. abortus* S19 TnSeq libraries. A dashed line indicates the ideal model where no difference between conditions is observed, while the blue lines represent the model fit to the effective density data. Genes that did not distribute proportionally to the fitted line were identified as outliers using Cook’s distance outlier analysis. The outliers (cut-off >4/total number of genes) are influential on the regression and are labeled in red. Influential genes indicate a fitness advantage or disadvantage when mutated in the two compared conditions. Expanded data related to panels (**B–E**) can be found on FigShare as referenced in Data Availability.

Illumina libraries were prepared from libraries 1–7 using an adapted protocol for the Illumina DNA Prep kit (https://dx.doi.org/10.17504/protocols.io.5qpvo9ewdv4o/v1). DNA was quantified with a Thermo Scientific Nanodrop 2000c and diluted to 500 ng in nuclease-free H_2_O. Libraries were tagmented with the bead-linked Tn5 transposase to fractionate the genomic DNA and ligate Illumina adaptors. Tagmented libraries underwent three rounds of nested PCR to amplify the transposon-containing fragments (Fig. 1A). The final Illumina libraries were quantified with a Qubit fluorometer (Invitrogen) using the Qubit HS dsDNA assay kit, and integrity was checked with the Fragment Analyzer automated electrophoresis (Agilent Technologies). Each library was loaded onto the Illumina NovaSeq X Plus S4 Flow Cell – PE 150 to generate 150 bp paired-end reads. Methodology on bioinformatic banalyses can be found at https://dx.doi.org/10.17504/protocols.io.81wgbrjwnlpk/v1.

Table 1 and Fig. 1A and B summarize the conditions used to generate TnSeq libraries and the sequencing results. TnDivA (6) was used to determine transposon insertion effective densities, enabling multiple statistical comparisons of Tn richness in highly saturated Tn libraries across conditions (Fig. 1C). For example, log2 fold change of effective density ratios revealed conditionally essential genes (Fig. 1D). Effective densities of each gene were plotted using a linear regression model to compare conditions, followed by Cook’s distance outlier analysis to identify genes whose Tn effective densities influence the regression (Fig. 1E). This rich data set can be used to explore genetic fitness of the pathogen *B. abortus* within and across multiple conditions.

## Data Availability

Additional methods are available on https://www.protocols.io/ and extended data of Tn reads, effective density, and statistical results for all TnSeq libraries are available on Figshare. This transposon sequencing project has been deposited in the NCBI Sequence Read Archive under BioProject accession number PRJNA1200015. The associated seven SRA numbers are SRX27245738–SRX27245743 and SRX27245745.
